# Comment “On the role of data, statistics and decisions in a pandemic” by Jahn et al.

**DOI:** 10.1007/s10182-022-00451-x

**Published:** 2022-06-18

**Authors:** Michael Höhle

**Affiliations:** Department of Mathematics, Stockholm University, 10691 Stockholm, Sweden

## Abstract

We comment the paper by Jahn et al. (On the role of data, statistics and decisions in a pandemic, 2022).

## Introduction

2.5 years after the first reported infections with Sars-CoV-2 a total of 6.3 million COVID-19 associated deaths have been reported. The COVID-19 pandemic is thus a reminder of the destructive impact infectious diseases can have. Although the pandemic has changed its character now that safe and effective vaccines have become available, low rates of vaccination, waning immunity and the emergence of new Sars-CoV-2 variants keep COVID-19 on the agenda.

Continuous preparation for the next pandemic during “peace-time” is the key for a better pandemic response. The work by Jahn et al. (2022) provides an overview on what role statistics can play in these preparations and how to improve matters from a statistical perspective. Focus of this comment is to review some of the authors’ suggestions relating to the population level using the situation in Germany as an example.

## Data availability and quality

The Protection Against Infection Act (IfSG) reporting data has been the main source used to take action during the pandemic in Germany. However, a number of additional data sources were generated or tapped to provide more reliable, less-biased and more timely insights. This ranges from population survey register data containing aggregated information on all ICU patients (Deutsche Interdisziplinäre Intensiv- und Notfallmedizin 2020), dedicated seroprevalence studies to more ad hoc “data-donation” projects based on convenience samples of data collected by fitness tracking devices (Robert Koch Institute 2022). Tapping these sources is important, but Jahn et al. (2022) make the point of checking each source against the eight criteria of the European Statistics Code of Practice. While each source is likely to fail one or several of these criteria, “timeliness” and “accuracy” are often contradictions, of which in the early phases of a pandemic “timeliness” usually wins. At the current stage of the pandemic, this trade-off should not have to be made the same way: However, for critical areas such as disease incidence, vaccinations or hospitalisations, Jahn et al. (2022) point out that the current data collecting procedures have not been (sufficiently) improved.

## From data to insights: the purposes of modelling

Models in the initial phase of a pandemic will be more expert knowledge based than data based. An example is the risk classification algorithm of the German Corona Warn App (CWA). It was developed purely on a mechanistic view of what the process from exposure to reporting of a positive test in the app looks like with parameters inferred from the literature, expert knowledge and informed guessing (Corona Warn App Team 2020; Meyer et al. 2021). The existence of such a generally accepted concept relying on the promise of anonymity with a statistical classifier at heart, downloaded more than 45 mio times, can be seen as a statistical success. However, the trick is to simultaneously plan how to collect data to verify these assumptions and continuously update model components and tuning parameters once launched.

Another important topic is the computation and interpretation of excess mortality: Unlike dynamic epidemic SIR-type modelling, the comparison of mortality has a long tradition in demographics and statistics with tools ranging from classical standardisation approaches (to adjust for age heterogeneity in the populations) to more sophisticated time-series-oriented generalized additive model approaches with penalized spline components (World Health Organisation 2022). It is surprising how much the choice of statistical method impacts the final results (De Nicola et al. 2022). However, a lack of agreed standards does not justify the use of simplistic models without age-adjustments. Instead, statistical institutions and professional scientific societies need to co-operate to establishing methodological standards.

## Communication

The authors stress how important basic data and statistical literacy of the recipients are for a successful communication. An additional example of this is the communication on the success of COVID-19 vaccines. This includes making the low risk of side effects perceivable to the individual or explaining the concept of baseline-fallacy when concerns arise that “half of COVID-19 hospitalised patients are vaccinated”.

Finally, the role of data journalists during the pandemic could be more appreciated. They have been important multipliers for communicating statistical results to the public - filling a gap of receiver-oriented communication by the institutions generating these data and statistical results. On the positive side, with quantitatively skilled data journalists as part of the communication process, transparency in terms of data, methods and code has become an important factor well beyond the scientific bubble. This is a promising development and appreciates that statistical solutions for complex problems cannot always be simple.

## Data Availability

Not relevant.

## Code availability

Not relevant.
